# Pre-reimbursement: early assessment for coverage decisions

**DOI:** 10.1007/s10354-019-0683-1

**Published:** 2019-02-06

**Authors:** Nicole Grössmann, Sarah Wolf, Katharina Rosian, Claudia Wild

**Affiliations:** 1 Ludwig Boltzmann Institute for Health Technology Assessment, Garnisongasse 7/20, 1090 Vienna, Austria; 2 Department of Health Economics, Center for Public Health, Medical University of Vienna, Vienna, Austria

**Keywords:** Reimbursement, Early assessment, Decision support, Health Technology Assessment, Health technologies, Erstattung, Frühbewertung, Entscheidungsunterstützung, Health Technology Assessment, Gesundheitstechnologien

## Abstract

**Background:**

In the past decade, the Ludwig Boltzmann Institute for Health Technology Assessment (LBI-HTA) has introduced two programs: “Horizon Scanning in Oncology” (HSO) and extra medical services (“MELs”), which are to facilitate coverage decisions based on early assessments. This article aims to outline the general process and methods within these two programs.

**Methods:**

A narrative-descriptive synthesis of the literature was performed to outline the general and LBI-HTA-specific processes and methods of early assessments.

**Results:**

In total, 79 HSO assessments (2009–2018) and 95 MELs (2008–2018) have been conducted by the LBI-HTA. Recently, additional methods that contribute to European applicability have been introduced into these programs.

**Conclusions:**

Overall, pre-coverage decisions based on early assessment reports are dependent on the existing evidence. However, the organisation of the health care system and the cross-linking between decision-makers and HTA institutions can have an impact.

## Zusammenfassung

**Grundlagen:**

Im letzten Jahrzehnt wurden am Ludwig Boltzmann Institut für Health Technology Assessment (LBI-HTA) 2 Programmlinien eingeführt, die auf der Grundlage von Frühbewertungen A‑priori-Erstattungsentscheidungen unterstützen sollen: „Horizon Scanning in Oncology“ (HSO) und medizinische Einzelleistungen (MEL). Ziel dieses Artikels ist es, allgemeine Prozesse und Methoden dieser Programmlinien darzustellen.

**Methodik:**

Eine narrativ-deskriptive Synthese der Literatur wurde durchgeführt, um sowohl generische als auch LBI-HTA-spezifische Prozesse und Methoden zu erläutern.

**Ergebnisse:**

Insgesamt wurden 79 HSO- (2009–2018) und 95 MEL-Berichte (2008–2018) vom LBI-HTA erarbeitet. Um eine europäische Zusammenarbeit zu ermöglichen und zu fördern, wurden kürzlich ergänzende Methoden in den Programmlinien eingeführt.

**Schlussfolgerungen:**

Insgesamt sind Frühbewertungen für A‑priori-Erstattungsentscheidungen abhängig von der verfügbaren Evidenz. Jedoch kann sowohl die Organisation des Gesundheitssystems als auch die Vernetzung zwischen Entscheidungsträgern und HTA-Institutionen einen Einfluss haben.

## Introduction

Health technology assessment (HTA) has gained increasing importance in supporting decisions. Over time, not only have specific HTA methodologies been developed, but the specific timing of assessments along the life-cycle of medical technologies has also evolved. Early assessments—before the broad diffusion of such health technologies—bear the advantage of supporting transparent, evidence-based reimbursement decisions on benefit catalogues, as well as of assisting budget allocations for costly medicinal products [1–3]. Depending on the developmental stage in the life-cycle of a health technology, different “early” HTA concepts like early dialogue (before the inception of clinical trials for market approval), early awareness systems or alert systems (for scanning the horizon of technologies in development or before market entry), and early (pre-coverage) assessments can be applied [1].

The fast implementation of new and often costly technologies into clinical practice and the high number of medicinal products in the pipeline have led to the introduction of early awareness systems, also called horizon scanning systems (HSS) [4–6]. Several countries like Sweden, Canada and the Netherlands have incorporated such concepts to make health care systems more effective and efficient through the early identification and prioritisation of emerging expensive and/or clinically relevant therapies [4, 7]. Austria ranks among the top ten countries when it comes to the rapid adoption and implementation of oncology drugs [8, 9]. Therefore, the Ludwig Boltzmann Institute for Health Technology Assessment (LBI-HTA) was commissioned in 2007 to develop and establish an HSS specifically focusing on anti-cancer drugs to provide a basis for better informed pre-coverage decisions [9].

Furthermore, many countries maintain benefit catalogues covering medical services and technologies that can be reimbursed. Some benefit catalogues cover all interventions of the in- and outpatient sectors, or—as in Austria—separate catalogues exist for the inpatient and outpatient sectors. In addition to the flat-rate payments based on a diagnosis-related group (DRG) system, costly interventions are reimbursed via a supplementary tariff in Austria. Within this hospital benefit catalogue, highly specialised services, such as medical devices and some specific oncology drugs, are offset as extra medical services (MELs) [10, 11]. Since 2008, the LBI-HTA has been conducting annual HTA reports (evidence syntheses) to support evidence-based, pre-coverage decisions on whether to include new interventions in the Austrian hospital benefit catalogue [11, 12].

This article aims to outline the general process of pre-coverage decision support and the scientific methods applied for early assessments on the basis of two routine programs implemented in Austria: Horizon Scanning in Oncology (HSO) and extra medical services (“MELs”).

## Materials and methods

To outline the general process of pre-coverage decision support with regard to LBI-HTA-specific methods also on an international level, a narrative-descriptive synthesis of the literature was performed. Therefore, Austrian and international HTA standards of early assessments are incorporated into this article. In addition to a targeted manual search, an internal methods handbook of the LBI-HTA was used as a basis to provide an overview of the methods and processes of early assessments in Austria [13]. Basically, both the literature search and selection always followed a consensus-led process in which three researchers (NG, KR/SW) were involved. Differences were resolved through discussion and consensus.

## Results

### Examples of two early assessment programs

#### Horizon Scanning in Oncology program—introduction and aim

As mentioned, Austria is one of the countries where new anti-cancer treatments are adopted faster and are available earlier after approval by the European Medicines Agency (EMA) compared to many other European countries [8, 9]. Therefore, the LBI-HTA was asked by regional hospital providers and the Austrian Ministry of Health (MoH) to establish an early awareness and alert system specifically focusing on cancer drugs. The so-called Horizon Scanning in Oncology (HSO) program was developed and tested between 2007 and 2008 in cooperation with horizon scanning experts from the EuroScan International Network (International Information Network on new or emerging, appropriate use and re-assessment needed Health Technologies). In October 2009, the program was routinely introduced. Since then, 83 LBI-HTA assessments and three updates have been conducted and published on the LBI-HTA website (http://eprints.hta.lbg.ac.at/view/types/dsd-hso.html). Two to three new therapies are assessed quarterly, resulting in at least eight early assessments annually. The aim of the HSO program is to identify new anti-cancer drugs or already approved drugs with licensing extensions that have a potentially relevant therapeutic and/or financial impact on the Austrian health care system. Thus, it shall not only contribute to a rational and evidence-based decision-making process, but also facilitate estimations of the impact on the health budget [8].

#### Horizon Scanning in Oncology program—process and method

**Fig. 1:**
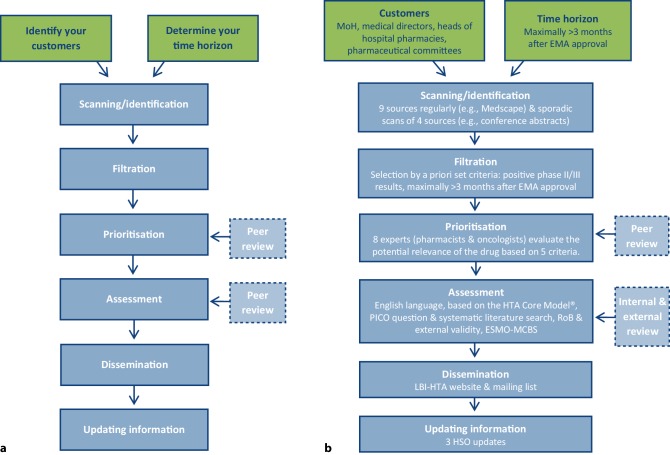
Steps of horizon scanning systems. **a** Generic process of horizon scanning systems. **b** Inherent steps of the Austrian Horizon Scanning in Oncology program. Adapted from: “A toolkit for the identification and assessment of new and emerging health technologies” [36]. *EMA* European Medicines Agency, *ESMO-MCBS* European Society of Medical Oncology-Magnitude of Clinical Benefit Scale, *LBI-HTA* Ludwig Boltzmann Institute for Health Technology Assessment, *MoH* Ministry of Health, *PICO* Population, Intervention, Comparator intervention(s) and patient-relevant Outcomes, *RoB* risk of bias

Across those countries that have implemented HSS, like the UK, Canada and Sweden, the inherent process basically includes the same steps (Fig. 1a; [6]). However, individual adaptations to respective contexts and needs, such as the time horizon of the HSS—which can vary from an early stage (phase I) to later stages (phase III) of drug development—have to be applied. In the HSO program of the LBI-HTA, a maximum time horizon of 3 months after marketing authorisation by the EMA, which mostly includes phase III trials, was chosen. As a first step, potential customers have to be determined prior to an HSS establishment. Thus, the LBI-HSO program was commissioned by the Austrian inpatient sector and should inform representatives of the Ministry of Health (MoH), medical directors, heads of hospital pharmacies and members of regional pharmaceutical committees [8].

The first step in the Austrian HSO process (Fig. 1b) is to identify only originator cancer drugs and indication extensions in development by regularly scanning nine sources (e.g., Medscape, websites of regulatory bodies) and periodically scanning four additional sources (e.g., conference abstracts). As a next step, drugs of the horizon scanning list that meet the inclusion criteria, which currently consists of over 400 cancer drugs in the development pipeline, are filtered out quarterly. Two blinded researchers make a first selection through a priori set filter criteria (about ten to fourteen), which mainly include the availability of positive phase III or phase II (orphan drugs) results and the maximum period of 3 months after EMA approval. Available information on the drug, indication, incidence, regulatory status and trial results are collected and sent quarterly to a group of eight experts made up of oncologists and hospital pharmacists. On the basis of five criteria, such as the potential for a significant health benefit to the patient group, the expert panel is asked to prioritise the drugs by choosing one of the following three categories—“highly relevant”, “relevant” and “not relevant”—to identify oncology drugs with a potential health and/or budget impact (Table 1). Based on the cross-sectional score of all experts, those drugs considered as highly relevant are selected for an assessment [8]. Results of the prioritisation are available on the LBI-HTA website (http://eprints.hta.lbg.ac.at/view/types/dsd-hso.html).

**Table 1:** Defined prioritisation criteria for the clinical expert panel of the Horizon Scanning in Oncology (HSO) program

Are there already other treatment regimen(s) available for this specific indication or is this drug a completely new therapy?	Treatment available New therapy
Will the new drug replace a current drug regimen or is it an add-on therapy for this indication?	Add-on Replace New therapy
Is there potential for a significant health benefit to the patient group (high clinical impact)?	Minor Major
Is there potential for a significant impact on drug budgets if the technology diffuses widely (because of expected moderate to high unit costs and/or because of high patient numbers)?	Minor Major
Is there potential for inappropriate use (off-label) of the technology?	Minor Major
Choose category	Highly relevant—assessment Relevant—monitor the drugs Not relevant—drop drug

Since 2015, the structure of HSO assessments has been based on the HTA Core Model® for Rapid Relative Effectiveness Assessment of Pharmaceuticals developed by the European network for HTA (EUnetHTA) [14]. The HTA Core Model® organises relevant information according to pre-defined generic research questions and clusters them into the following five domains: description of the technology, health problem and current use, clinical effectiveness and safety. In addition to the HTA Core Model®, chapters regarding the systematic literature search, clinical effectiveness and safety of further studies, ongoing trials, costs (ex-factory prices) and a comprehensive commentary section are implemented [8]. As a first step in conducting an HSO assessment, the research question has to be defined and a systematic literature search has to be performed in relevant databases like PubMed and Embase. The reports are written in English so that they may also be used in other European health care systems and to reduce redundancies in European HTA [15].

To facilitate further European collaboration and to continuously refine scientific methods, additional research tools have been incorporated into the HSO reports in recent years. Therefore, the internal and external validity of clinical trials are assessed by applying tools based on EUnetHTA guidelines. These methods assess the methodological quality of evidence by evaluating the risk of bias (RoB) at study level (internal validity) and the applicability of study results, specifically the extent to which the results can be generalised to clinical practice (external validity) [16, 17]. Moreover, the Magnitude of Clinical Benefit Scale (MCBS) developed by the European Society of Medical Oncology (ESMO) was introduced to systematically and transparently evaluate the magnitude of meaningful clinical benefit (MCB) that can be expected from a new cancer drug [5, 18, 19]. The scale can only be applied to solid tumour drugs and ranges from zero to five, whereby scores of four and five indicate MCB. The evaluation of the clinical benefit is based on a dual rule taking both the observed relative benefit (hazard ratio) and the absolute benefit (median) into account. HSO assessments are internally and externally reviewed to guarantee quality assurance.

To ensure a transparent HSS system, final HSO reports are published on the LBI-HTA website (http://eprints.hta.lbg.ac.at/view/types/dsd-hso.html), as well as sent out via a mailing list including health stakeholders from different areas including oncologists, hospital pharmacists, medical directors, representatives of the MoH and health insurance providers [8]. Additionally, an HSS has to monitor upcoming evidence of already approved therapies and conduct assessment updates. Between 2009 and 2016, 63 novel cancer indications were under evaluation within the HSO program of the LBI-HTA and three assessments were updated later on; 60 of those were consequently approved by the EMA.

#### Extra medical services program—introduction and aim

**Fig. 2:**
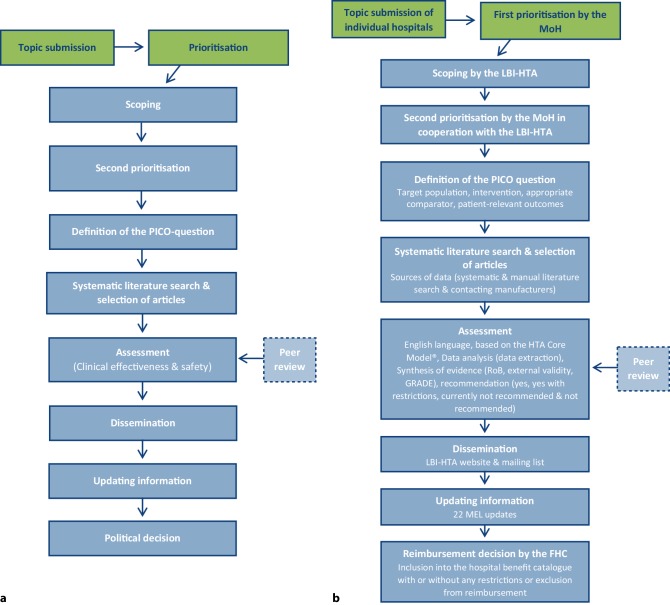
Work process of conducting early assessments. **a** Generic process of early assessments. **b** Inherent steps of the Austrian Extra Medical Services program. *GRADE* Grading of Recommendations Assessment, Development and Evaluation, *MoH* Ministry of Health, *LBI-HTA* Ludwig Boltzmann Institute for Health Technology Assessment, *PICO* Population, Intervention, Comparator intervention(s) and patient-relevant Outcomes, *RoB* risk of bias

As one of the first European countries, Austria began introducing a systematic evaluation of new hospital interventions submitted for reimbursement in 2008. The Federal Health Commission (FHC) of the Austrian MoH maintains a benefit catalogue of medical interventions that are provided in the inpatient sector. As mentioned in the introduction, the hospital benefit catalogue includes a list of MELs for which costs are reimbursed in addition to the DRG flat rates [11, 12]. Every year, suggestions for new interventions are submitted electronically by individual hospitals or by regional hospital cooperatives to the FHC at the MoH. These topics are compiled (in a long list) and subsequently prioritised by the MoH according to criteria such as the same suggestions by multiple hospitals or high-volume/high-cost suggestions. Finally, a (short) list of new interventions is prepared and the LBI-HTA is commissioned to conduct assessments. These topics are single-technology assessments (STA; one device/intervention for one indication) or multiple-technology assessments (MTA; one device/intervention for several indications or several interventions for one indication). The evidence syntheses have to be conducted within 3–5 months [12]. These assessments serve as evidence-based decision-support documents that include a recommendation to the FHC [11, 12]. These systematic evaluations follow generic steps that are represented in Fig. 2a. The introduction of this process was accompanied by the implementation of a new decisional option, the “XN code”. In contrast to preceding years when the sole options were to include/exclude interventions in the benefit catalogue and were not based upon evidence, XN interventions can be included temporarily (3–5 years) and will be re-evaluated [20]. Within the period of 2008–2018, 96 MELs (74 new systematic reviews and 22 updates) were carried out by the LBI-HTA (http://eprints.hta.lbg.ac.at/view/types/dsd.html).

#### Extra medical services program—process and methods

After initial compilation and prioritisation by the MoH, potentially relevant topics are provisionally communicated to the LBI-HTA for scoping. This process includes the scanning of different sources, like the HTA and Planned and Ongoing Projects (POP) Databases, Deximed, UpToDate, S3 guidelines (*Arbeitsgemeinschaft der Wissenschaftlichen Medizinischen Fachgesellschaften*, AWMF), other evidence-based guidelines and Open Knowledge Maps [15]. These consist of information from other evidence-based advisory boards (e.g., The National Institute for Health and Care Excellence, NICE; or the Canadian Agency for Drugs and Technologies in Health, CADTH). High-quality systematic reviews or assessments of other institutions and information on the regulatory and implementation status of the technology can thereby be identified. Accordingly, there is a second prioritisation by the MoH in cooperation with the LBI-HTA when the decisions on the final topics for the ensuing assessments are made (Fig. 2b).

After the final selection of the topics, the exact research question is defined by clarifying the target *P*opulation, the investigated *I*ntervention, the *C*omparator intervention(s) and patient-relevant *O*utcomes (PICO). A systematic literature search is then conducted in the following databases: Medline via Ovid (including PubMed), Embase, The Cochrane Library, and the Centre for Reviews and Dissemination (CRD). Furthermore, to identify ongoing and unpublished studies, a search in three clinical trial registries (ClinicalTrials.gov, WHO-ICTRP, EU Clinical Trials) is carried out. As a final step of the systematic literature search, a PRISMA (Preferred Reporting Items for Systematic Reviews and Meta-Analyses) flow diagram has to be prepared and implemented in the assessment [13, 21].

Subsequently, the quality of the studies is rated by an RoB assessment. Based on the study design, different methodological tools can be considered for the RoB assessment (e. g., Cochrane RoB checklist, ROBINS-I, AMSTAR, IHE 20-Criteria Checklist). The external validity is additionally evaluated, i. e., the extent to which the results can be generalised to the population in clinical practice. In a next step, efficacy and safety data of the included studies are extracted by one author and reviewed by another independent author [16, 22]. Since 2016, the structure of MEL assessments has been based on the HTA Core Model® (Application for Rapid Relative Effectiveness Assessment), which was developed within EUnetHTA (www.eunethta.eu, see Horizon Scanning in Oncology program—process and method) [23].

Accordingly, a critical appraisal based on the Grading of Recommendations Assessment, Development and Evaluation (GRADE) framework is conducted to evaluate whether the overall strength of evidence supports the conclusions [24]. Therefore, the extracted outcomes have to be categorised, according to patient relevance, as either decisive for the final decision (critical), clinically important (important) or not clinically important (not important). To estimate the effect for each outcome, data on selected outcome categories are synthesised across studies by two independent researchers in a summary of findings table [24]. To rank the overall strength of evidence across all outcomes, GRADE applies four categories, whereby the lowest quality of any critical outcome is decisive (Table 2). The main focus is on the predefined outcomes of the PICO question and the outcomes defined as critical during the GRADE process. On these grounds, conclusions are derived and recommendations are given on whether the new hospital intervention should be included in the MEL list and thus reimbursed.

**Table 2:** Categories to rank the overall strength of evidence based on Grading of Recommendations Assessment, Development and Evaluation (GRADE) [24]

Strength of evidence	Description
High	We are very confident that the true effect lies close to that of the estimate of the effect
Moderate	We are moderately confident in the effect estimate: the true effect is likely to be close to the estimate of the effect, but there is a possibility that it is substantially different
Low	Our confidence in the effect estimate is limited: the true effect may be substantially different from the estimate of the effect
Very low	Evidence either is unavailable or does not permit a conclusion

The recommendation can include one of the four following categories: “yes”, “yes—with restrictions”, “no—preliminary” and “no” (Table 3). The categories “yes” and “no” shall recommend or not recommend the inclusion into the hospital benefit catalogue, respectively. Moreover, a “recommendation with limitations” includes a statement on advised restrictions (e. g., use in specialised centres). Regarding the third category of “preliminary rejection”, the recommendation is accompanied by a description of gaps in clinical evidence (e. g., lack of long-term follow-up data or controlled studies, unreliable clinical endpoints). LBI-HTA recommendations given in the MEL assessments are taken into consideration by the FHC. However, the final decision of the FHC can be either the inclusion into the hospital benefit catalogue with or without any restrictions or the exclusion from reimbursement [12]. After amendments to the hospital benefit catalogue have been published, early assessments are published via the LBI-HTA website (http://eprints.hta.lbg.ac.at/view/types/dsd.html) and a mailing list addressing health care stakeholders.

**Table 3:** Definitions and appraisal results of pre-coverage recommendations of the Ludwig Boltzmann Institute for Health Technology Assessment (LBI-HTA) and final decisions of the Federal Health Commission (*n* = 69, 2008–2017)

Variable	Category	Definition	Assessment, *n* (%)
Recommendation	Yes	The inclusion in the catalogue of benefits is recommended	0 (0.0)
	Yes (with restrictions)	The inclusion in the catalogue of benefits is recommended with restrictions	15 (21.7)
	No (preliminary)	The inclusion in the catalogue of benefits is currently not recommended	50 (72.5)
	No	The inclusion in the catalogue of benefits is not recommended	4 (5.8)
Decision	Yes	The coverage is accepted and the technology is included in the MEL list	5 (7.2)
	Yes (with restrictions)	The intervention is included in the hospital benefit catalogue via special coding “XN code” (with restrictions), thus, the procedure has to be reassessed within a defined time frame	20 (29.0)
	No	The intervention is not included in the MEL list	44 (63.8)

Between 2008 and 2017, 69 assessments for hospital interventions (often, but not always medical devices) that can be followed-up to a political decision were conducted within the MEL program of the LBI-HTA. Twenty-five (36.2%) of those were included into the hospital benefit catalogue, whereby only five (7.2%) were included without any restrictions. On the other hand, 44 (63.8%) were not eligible for inclusion into the catalogue. Regarding the MEL recommendations, 54 (78.3%) interventions were initially not recommended to be included, of which 50 (72.5%) were preliminarily not recommended due to gaps in clinical evidence. Fifteen (21.7%) of the interventions were recommended with restrictions for inclusion into the benefit catalogue and none were recommended without any restrictions (Table 3).

## Discussion

Many countries have implemented HTA concepts to forecast the benefit–risk ratio of costly interventions in order to support transparent, evidence-based health care reimbursement decisions, but also to facilitate budget allocations [1, 12, 25]. Thus, decision support can either be directly linked to political decisions, e. g., directly by shaping the hospital benefit catalogue (MEL) or indirectly by facilitating decentralised drug procurement decisions, e. g., for drug commissions of hospital owner organisations (HSO). Therefore, this study sought to address the inherent process and methods of pre-coverage decision support of early assessments in Austria, based on the HSO and the MEL program of the LBI-HTA. Our findings indicate that the recommendations made in our MEL reports have been directly translated into reimbursement decisions in the majority of instances. More than half of the evaluated MELs were not initially recommended and consequently not included in the Austrian hospital benefit catalogue. Regarding the HSO program, no conclusion on the application of early assessments can be drawn. One reason is the rather complex and fragmented Austrian health system, especially in the inpatient sector. On the other hand, our HSO program is rather reactive (evaluating identified costly drugs) and not directly linked to initial political requests. However, there is evidence that HSO assessments and the incorporated ESMO-MCBS scores contribute to decisions of Austrian hospital providers to some extent.

There are an increasing number of fast track approval pathways accompanied by the approval of drugs with ambiguous benefit–risk profiles [9, 26–29]. Since the bar for drug approval will most probably not be raised again, health care decision-makers have to continually assess and monitor the upcoming evidence of new and already approved cancer drugs [5]. Early awareness systems and HSS have thus become increasingly important in recent years. Therefore, the LBI-HTA carried out three updates of previously assessed therapies within its HSO program and conducted a cross-sectional study on the knowledge of cancer drugs at the time of approval [9]. In these studies, we were able to show that in a large number of therapies, no valid knowledge about the survival benefit is available at the time of marketing authorisation. Therefore, we are currently working on a follow-up study to monitor and characterise recently approved therapies with ambiguous benefit–risk profiles and identify any post-approval updates on survival and quality of life. In addition, the ESMO-MCBS was implemented to promote a transparent and standardised cancer drug evaluation process. By recommending medicines with a clinical benefit for reimbursement, scarce health care resources are allocated in a more efficient way [18, 19].

In contrast to pharmaceuticals, recommendations in early assessments of new hospital interventions are even more limited by the quality of available clinical evidence [12, 30]. Since it has been shown that the MEL assessments of the LBI-HTA are often not supported by strong evidence (randomised controlled trials), making recommendations can be challenging [12]. Moreover, the risk class of a medical device has an influence on issued decisions, as higher-risk procedures (e. g., invasive surgery) are usually not underpinned by strong evidence; on the contrary, they are associated with greater uncertainties. In Austria, despite limited evidence, a high risk profile often leads to an inclusion into the hospital benefit catalogue. This could be due to ethical considerations, like in the case of radiofrequency ablation in patients with painful vertebral metastases. This intervention provides pain relief and causes no major complications in a fatal disease accompanied by intolerable pain, and may thus be justified on ethical grounds [31, 32]. Continuous improvement of pre-coverage assessments through the implementation of new, transparent and standardised methods like GRADE is of high importance. Furthermore, changes at a regulatory level will be needed to ensure approvals of interventions with reliable benefit–risk ratios and strong evidence [11, 12].

European collaborations regarding HTA reports and HSS have gained importance in the past two decades [15, 25, 33]. Several collaborations on these topics have evolved, like EUnetHTA, BeNeLuxA, the International Network of Agencies for Health Technology Assessment (INATHA) and EuroScan. These networks shall not only help to efficiently allocate scarce resources and support pre-coverage decisions, but also to avoid redundancies [25]. A recent publication [34] has shown that the number of duplicate reports on technologies ranges from seven to 22. This leaves much room for improvement with regards to efficient cooperation between EU countries. Therefore, the HTA Core Model® was implemented in the early assessment procedure of the LBI-HTA, enabling a structured, consistent and easily transferable way to facilitate sharing information at an international level [23, 25, 35]. To remove language barriers, our early assessments are conducted in English, but German margins and summaries are available to guarantee national decision support. Austria has shown itself to be a forerunner in the systematic evaluation of hospital interventions and therefore plays a major role in the European coordination of collaboration on medical devices and interventions.

## Conclusion

Overall, coverage decisions based on early assessment reports not only depend on the available evidence, but are also influenced by the organisation of the health care system and the cross-linking between decision-makers and HTA institutions. Since the early assessments of the MEL program of the LBI-HTA are directly linked to the maintenance and shaping process of the hospital benefit catalogue covering the inclusion or exclusion of new technologies, they have an actual impact on reimbursement decisions and encourage more transparent and evidence-based decisions. Therefore, the use of HTA has become more prospective and proactive rather than solely reactive. In contrast, HSS could facilitate decentralised medical procurement decisions by supporting, for example, drug commissions of hospital owner organisations. Although the HSO program is efficient in identifying new potential indications for cancer drugs, the extent of the actual implication of these assessments on pre-coverage decisions remains unclear. Overall, the article offers a comparison of Austrian early assessments including international methodological standards and a general overview of two different approaches to decision support on costly drugs and medical devices.
